# The Impact of Major and Minor Phytocannabinoids on the Maintenance and Function of INS-1 β-Cells Under High-Glucose and High-Lipid Conditions

**DOI:** 10.3390/molecules30091991

**Published:** 2025-04-30

**Authors:** Esmaeel Ghasemi Gojani, Bo Wang, Dong-Ping Li, Olga Kovalchuk, Igor Kovalchuk

**Affiliations:** Department of Biological Sciences, University of Lethbridge, Lethbridge, AB T1K 3M4, Canada; esmaeel.ghasemigojan@uleth.ca (E.G.G.); bo.wang5@uleth.ca (B.W.); dongping.li@uleth.ca (D.-P.L.)

**Keywords:** type 2 diabetes, delta-9-tertrahydrocannabinol, cannabidiol, delta-9-tetrahydrocannabivarin, cannabichromene, cannabidiol, apoptosis, glucose stimulated insulin secretion

## Abstract

Type 2 diabetes mellites (T2DM) is the most common form of diabetes and affects a significant portion of the population. Obesity-related increases in free fatty acids and glucose in the diet contribute to β-cell dysfunction and loss, ultimately leading to the onset of T2DM. The endocannabinoid system, which is present throughout the body, plays a vital role in regulating various physiological processes, including those in the pancreas. This system has been implicated in metabolic disorders like obesity and diabetes, as it helps to regulate appetite, food intake, and fat production. Phytocannabinoids from *Cannabis sativa* have the potential to influence the endocannabinoid system, offering a promising therapeutic approach for diabetes and its complications. Using high-glucose–high-lipid (HGHL)-induced INS-1 β-cells, we investigated the protective effects of two major (THC and CBD) and three minor (THCV, CBC, and CBG) phytocannabinoids on high glucose–high lipid (HGHL)-induced apoptosis, cell cycle disruption, and impaired function of beta-cells. Our results showed that all five phytocannabinoids reduced HGHL-induced apoptosis, likely by decreasing TXNIP protein levels. Additionally, THC and all three minor phytocannabinoids provided protective effects against functional impairments caused by HGHL exposure.

## 1. Introduction

The prevalence of type II diabetes mellitus (T2DM) is rising globally, with cases now appearing at younger ages, including children. This shift highlights the growing impact of risk factors such as excessive weight gain, reduced physical activity, and the influence of modern dietary patterns. Addressing this trend demands urgent attention to prevention strategies and healthier lifestyle choices to reduce the burden of T2DM on individuals and healthcare systems [1]. This chronic metabolic condition is defined by persistent hyperglycemia resulting from impaired insulin secretion and resistance. Alarmingly, the 2023 International Diabetes Federation (IDF) Diabetes Atlas reported that 537 million adults aged 20–79 were living with diabetes in 2021. This number is expected to escalate to 643 million by 2030 and 783 million by 2045 [2]. Representing over 90% of all diabetes cases, T2DM remains the predominant and most concerning form of the disease. Addressing this epidemic requires urgent action to mitigate its increasing burden [2]. The onset of T2DM begins with insulin resistance, where cells like hepatocytes, adipocytes, and muscle cells fail to respond effectively to insulin. This disrupts glucose and lipid regulation and triggers a compensatory increase in insulin production by β-cells. Over time, this excessive strain leads to glucolipotoxicity and inflammation [1,3]. Insulin resistance-driven glucolipotoxicity harms β-cells through mechanisms like **endoplasmic reticulum (ER)** stress and disrupted insulin signaling. These effects reduce glucose-stimulated insulin secretion (GSIS), promote apoptosis, and lead to β-cell loss. Persistent ER stress from heightened insulin production demands further worsens β-cell dysfunction and decline [4].

Glucolipotoxicity also contributes to β-cell dedifferentiation, a key driver of diabetes progression. In T2DM, β-cells lose their specialized functions, including insulin production, and begin to resemble other islet cell types, such as α- and δ-cells or progenitor-like states [4,5,6]. This transformation is triggered by changes in gene expression caused by oxidative stress, ER stress, and inflammation under conditions such as hyperglycemia and hyperlipidemia [7,8]. Critical transcription factors, such as PDX1, MafA, and NEUROD1, which are essential for maintaining β-cell identity, become dysregulated [9,10]. As a result, β-cell dysfunction and islet remodeling play a central role in the progression of diabetes.

*Cannabis sativa* produces a diverse array of phytocannabinoids that interact with the endocannabinoid system through both receptor-dependent and receptor-independent pathways, influencing various physiological and pathological processes. Among these compounds, delta-9-tetrahydrocannabinol (THC) and cannabidiol (CBD) are the most extensively studied cannabinoids due to their broad therapeutic potential. THC, known for its psychoactive effects, has demonstrated anti-inflammatory and analgesic properties, while CBD has shown promise in reducing inflammation, oxidative stress, and neurodegeneration. In addition to the major cannabinoids, minor phytocannabinoids such as tetrahydrocannabivarin (THCV), cannabichromene (CBC), and cannabigerol (CBG) have emerged as potential therapeutic agents. These minor cannabinoids exhibit unique pharmacological profiles, targeting various components of the endocannabinoid system and showing potential in managing inflammatory and metabolic disorders, including T2DM. For example, THCV has been shown to improve glucose metabolism and insulin sensitivity, while CBC and CBG exhibit anti-inflammatory and cytoprotective effects [11].

Despite these promising findings, the role of phytocannabinoids in T2DM remains underexplored, particularly concerning their effects on the critical stages of disease progression. These stages include β-cell dysfunction, characterized by impaired GSIS, β-cell loss due to glucolipotoxicity-induced apoptosis, and β-cell dedifferentiation. Current research has provided only a fragmented understanding of how major and minor phytocannabinoids influence these interconnected pathways. To address these gaps, our study investigates the effects of five selected major and minor phytocannabinoids on β-cell viability, GSIS, and dedifferentiation using an in vitro model. This comprehensive analysis aims to uncover the therapeutic potential of these phytocannabinoids in mitigating β-cell dysfunction and loss, thus offering new insights into their role in mitigating T2DM. By examining their mechanisms of action, our research seeks to advance the understanding of how these compounds could be used to modulate the endocannabinoid system and improve metabolic health.

## 2. Results

### 2.1. All Five Phytocannabinoids Mitigate HGHL-Induced Apoptosis in INS-1 β-Cells

**Figure 1:**
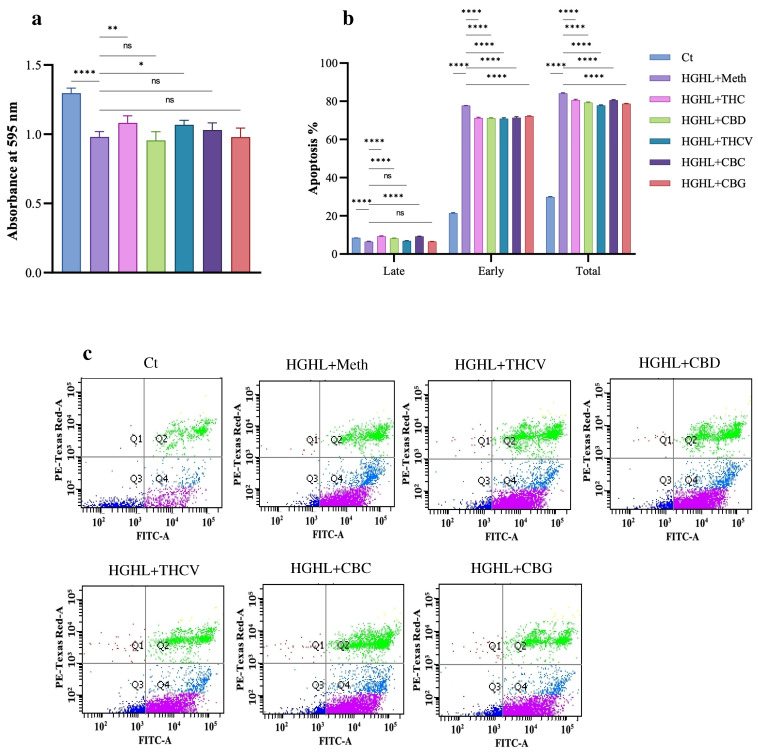

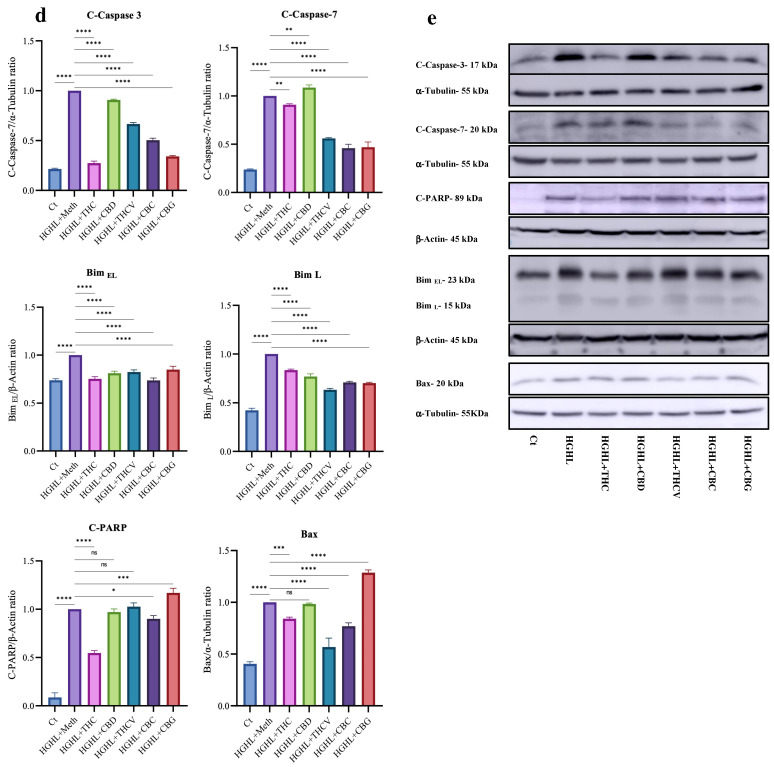
Results of the MTT assay, apoptosis assay, and Western blot analysis of apoptotic biomarkers. INS-1 β-cells were pretreated with each phytocannabinoid for 2 h, followed by exposure to HGHL conditions for 48 h. Treated cells were analyzed using the following methods: (**a**) MTT assay results showing the effects of 5 µM of each phytocannabinoid on the viability of HGHL-induced INS-1 β-cells. (**b**) Apoptosis assay results of INS-1 β-cells co-treated with phytocannabinoids under HGHL conditions. (**c**) Flow cytometry graphs displaying the four quadrants (Q1–Q4) of the apoptosis assay. Color map: Q3 (viable cells) → black dots (Annexin V−/PI−), Q4 (early apoptotic cells) → blue/purple dots (Annexin V+/PI−), Q2 (late apoptotic cells) → green dots (Annexin V+/PI+), and Q1 (necrotic cells) → pink dots (Annexin V−/PI+). (**d**) Western blot analysis of apoptotic biomarkers, including C-Caspase-3, C-Caspase-7, C-PARP, Bim _EL_, Bim _L_, and Bax, in response to 5 µM of each phytocannabinoid under HGHL conditions. (**e**) Western blot images of the apoptotic biomarkers. Abbreviations: Ct (control), HGHL (high glucose + high lipid) and Meth (Methanol). All data are presented as mean ± SD, *n* = 3 measurements. Asterisks indicate significant differences: * *p* < 0.05, ** *p* < 0.01, *** *p* < 0.001, and **** *p* < 0.0001; ns—non-significant.

To investigate the impact of phytocannabinoid co-treatment on the reduced viability of HGHL-induced INS-1 β-cells, an MTT assay was performed. The results revealed that co-treatment with 5 µM THC and THCV significantly restored the reduced viability of HGHL-induced INS-1 β-cells. In contrast, CBD, CBC, and CBG did not exhibit a significant positive effect on cell viability (Figure 1a). The apoptosis assay results showed that all five phytocannabinoids effectively mitigated HGHL-induced late and total apoptosis, the combined percentage of early apoptotic and late apoptotic/secondary necrotic cells (Annexin V+/PI− and Annexin V+/PI+) (Figure 1b,c). Additionally, the response of apoptotic biomarkers, including Cleaved-Caspase-3 (C-Caspase-3), Cleaved-Caspase-7 (C-Caspase-7), Cleaved-PARP (C-PARP), Bax, Bim EL, and Bim L, was examined. All five phytocannabinoids reduced the elevated levels of C-Caspase-3, Bim EL, and Bim L. THC, THCV, and CBC significantly decreased the levels of C-Caspase-7, while CBD exhibited a stimulatory effect on this caspase. THC and CBC mitigated the increased levels of C-PARP, whereas THCV and CBD had no significant effect on its levels. In contrast, co-treatment with CBG further elevated the level of C-PARP in HGHL-induced INS-1 β-cells. THC, THCV, and CBC reduced the elevated expression of the pro-apoptotic protein Bax, while CBG further increased its expression in HGHL-induced INS-1 β-cells. CBD, however, did not produce any significant change in Bax expression levels (Figure 1d,e).

### 2.2. Phytocannabinoids Alter the Distribution of Cells in the G1, S, and G2 Phases of the Cell Cycle in HGHL-Induced INS-1 β-Cells

**Figure 2:**
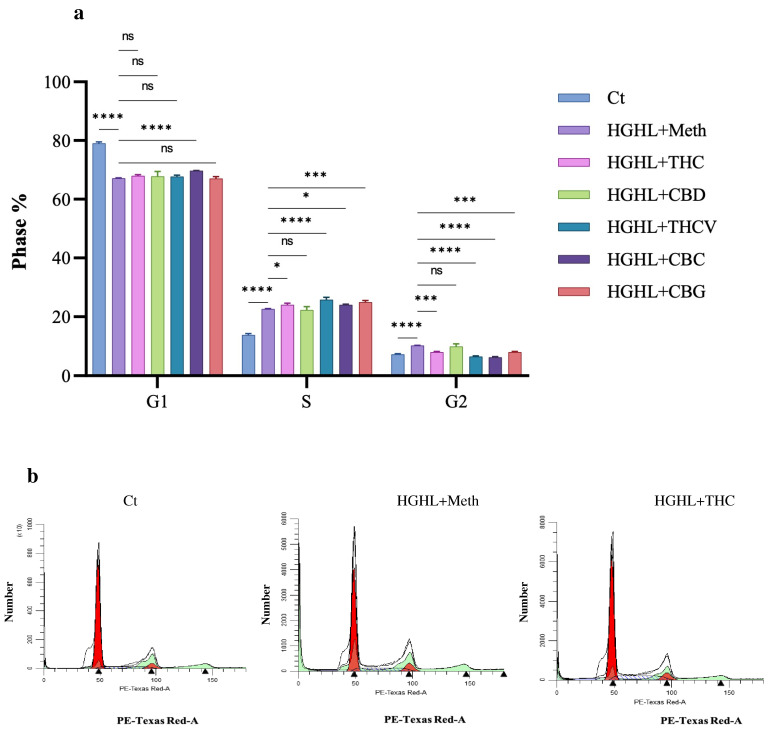

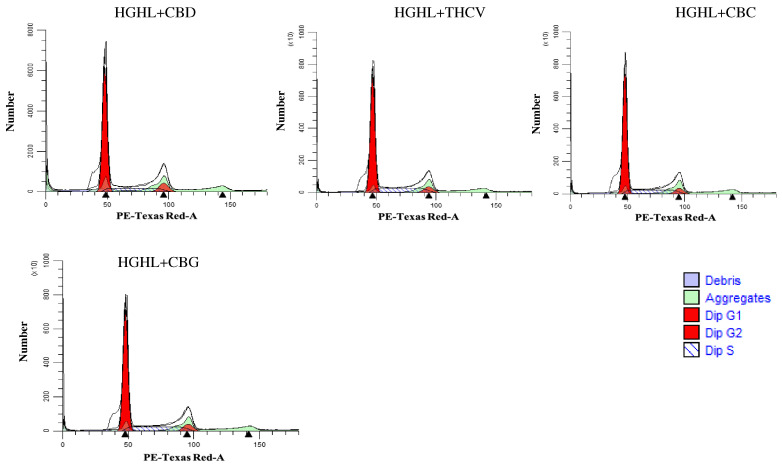
Cell cycle analysis of HG–HL-induced INS-1 β-cells treated with the phytocannabinoids. (**a**) Bar graphs show the percentage of cells in the G1, S, and G2 phases under HG–HL conditions following treatment with 5 µM THC, CBD, THCV, CBC, and CBG. (**b**) Representative histograms display the distribution of cells in the G1, S, and G2 phases. Peaks correspond to DNA content in different phases of the cell cycle, and shifts in these peaks indicate alterations in cell cycle progression. Abbreviations: Ct (control), HGHL (high glucose + high lipid), Meth (Methanol). Data are presented as mean ± SD, n = 3 measurements. Asterisks indicate significant differences: * *p* < 0.05, *** *p* < 0.001, and **** *p* < 0.0001; ns—non-significant.

According to our results, HGHL conditions reduce entry into the G1 phase while increasing entry into the S and G2 phases. CBC treatment was the only treatment to significantly restore the reduced level of G1. All phytocannabinoids, except CBD, further increased the percentage of cells in the S-phase in HGHL-induced INS–1 β-cells. In contrast, all phytocannabinoids, except CBD, reduced the elevated levels of G2-phase entry in HGHL-induced INS-1 β-cells (Figure 2).

### 2.3. All Phytocannabinoids Except CBD Restore Impaired GSIS in HGHL-Induced INS-1 β-Cells

**Figure 3:**
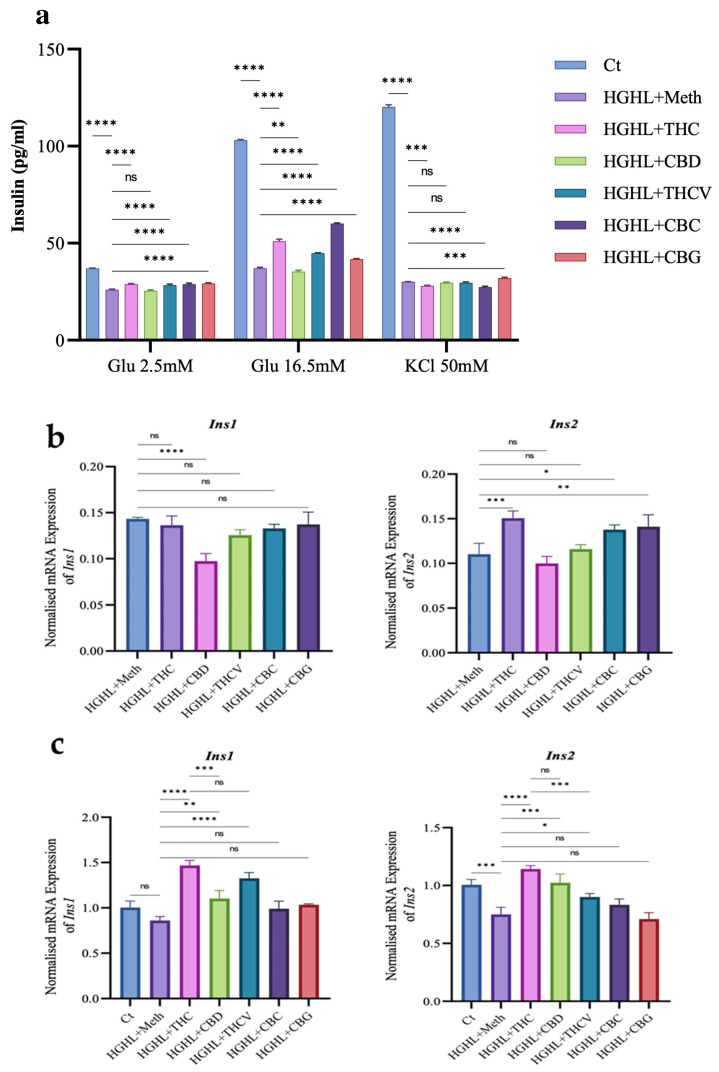
GSIS, KSIS, and insulin mRNA/protein production in response to each phytocannabinoid in HGHL-induced INS-1 β-cells. (**a**) GSIS and KSIS results in HGHL-induced *INS-1* β-cells treated with the phytocannabinoids. (**b**) q*RT*-PCR results of Ins genes in the co-treatment experiment in response to 5 µM of each phytocannabinoid. (**c**) qPCR results of Ins genes in the posttreatment experiment in response to 5 µM of each phytocannabinoid. Abbreviations: Ct (control), HGHL (high glucose + high lipid) and Meth (Methanol). Data are presented as mean ± SD, n = 3 measurements. Asterisks indicate significant differences: * *p* < 0.05, ** *p* < 0.01, *** *p* < 0.001, and **** *p* < 0.0001; ns—non-significant.

The effects of phytocannabinoids on HGHL-induced INS-1 β-cells were assessed through GSIS, KSIS, and insulin gene/protein expression analyses. THC, THCV, CBC, and CBG significantly restored the impaired GSIS, while CBD negatively impacted impaired GSIS. Similarly, for basal insulin secretion (at 2.5 mM glucose), THC, THCV, CBC, and CBG demonstrated restorative effects, with CBD showing no negative impact. In the KSIS assay, only CBG improved the impaired secretion activity, while CBD and THCV showed no significant effects, and THC and CBC reduced this activity (Figure 3a). Regarding insulin gene expression, qPCR results showed that THC, CBC, and CBG upregulated the Ins2 gene, while THCV and CBD had no effect. None of the phytocannabinoids restored the reduced expression of the Ins1 gene, and CBD further downregulated it (Figure 3b). In posttreatment experiments, THC, CBD, and THCV restored both insulin genes’ expression levels, while CBC and CBG had no significant impact (Figure 3c).

### 2.4. All Five Phytocannabinoids Mitigate Elevated TXNIP While Enhancing Reduced CB1R Expression

**Figure 4:**
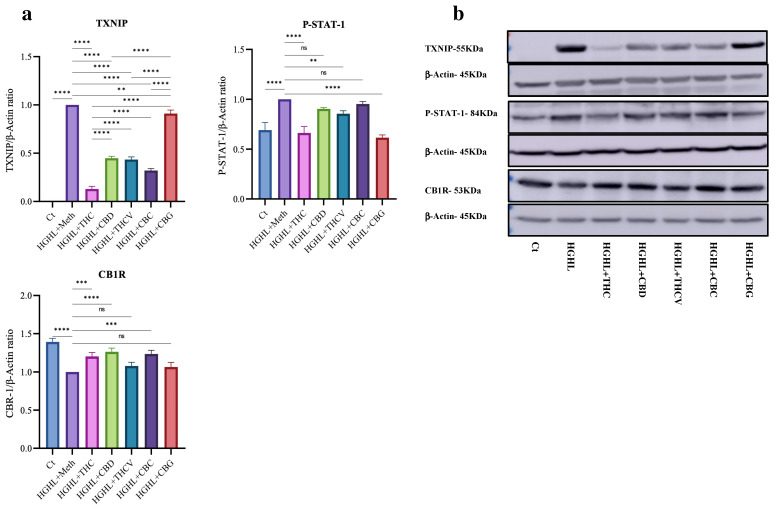
The Western blot analysis of TXNIP, P-STAT-1, and CB1R in HGHL-induced INS–1 β-cells in response the phytocannabinoids. (**a**) Quantitative analysis of TXNIP, P-STAT-1, and CB1R protein levels in response to the phytocannabinoids in HGHL-induced INS-1 β-cells. (**b**) Cropped Western blot images showing the bands of each target protein alongside their respective β-actin bands. Abbreviations: Ct (control), HGHL (high glucose + high lipid), and Meth (methanol). Data are presented as mean ± SD, n = 3 measurements. Asterisks indicate significant differences: ** *p* < 0.01, *** *p* < 0.001, and **** *p* < 0.0001; ns—non-significant.

This study also aimed to identify potential upstream targets influenced by the phytocannabinoids. Western blot analysis revealed that all five phytocannabinoids reduced the elevated levels of TXNIP in HGHL-induced INS-1 β-cells, with THC demonstrating a particularly pronounced modulatory effect. While the elevated levels of P-STAT-1 were mitigated by THC, THCV, and CBG, co-treatment with CBD and CBC did not significantly affect this transcription factor. Interestingly, HGHL conditions reduced the expression of the CB1R receptor in INS-1 β-cells. Co-treatment with THC, CBD, and CBC restored CB1R levels, whereas THCV and CBG showed no impact on this protein (Figure 4).

### 2.5. Certain Phytocannabinoids Positively Influence the Reduced Levels of β-Cell-Enriched Genes and Proteins

**Figure 5:**
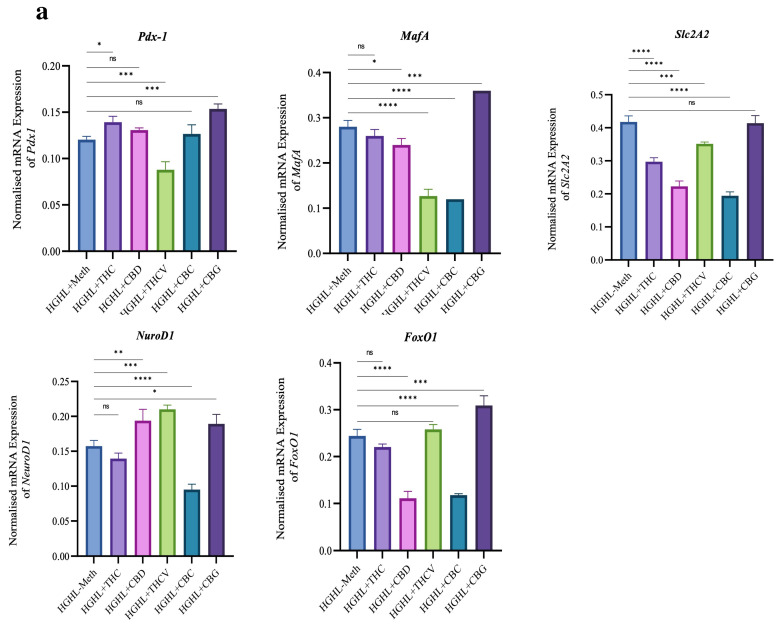

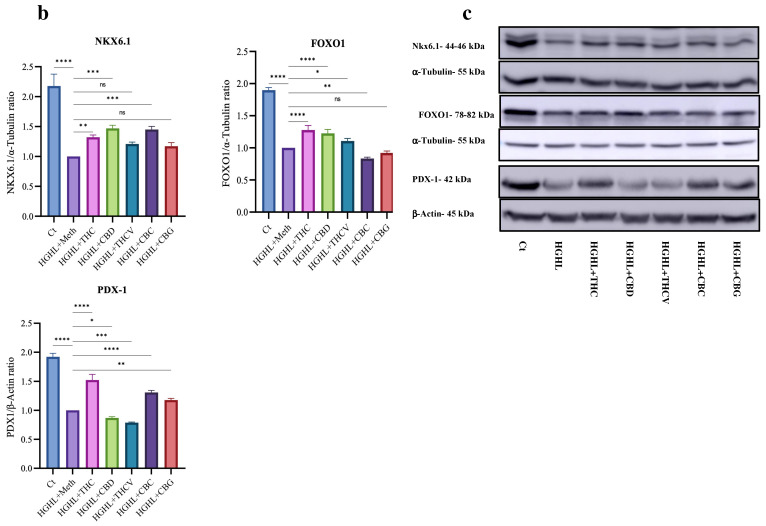
The Western blot and qRT-PCR analysis of β-cell-enriched genes and proteins in HGHL-induced INS–1 β-cells in response to the phytocannabinoids. (**a**) qRT-PCR analysis of *Pdx-1*, *MafA*, *Slc2A2*, *NeuroD1*, and *FoxO1* mRNAs in response to the phytocannabinoids in HGHL-induced INS–1 β-cells. (**b**) Western blot analysis of NKX6.1, FOXO1, and PDX-1 proteins in INS–1 β-cells co-treated with HGHL and 5 μM of each phytocannabinoid for 48 h. (**c**) Cropped Western blot images illustrating the target protein bands alongside their respective β-actin bands. Abbreviations: Ct (control), HGHL (high glucose + high lipid), and Meth (methanol). Data are presented as mean ± SD, n = 3 measurements. Asterisks indicate significant differences: * *p* < 0.05, ** *p* < 0.01, *** *p* < 0.001, and **** *p* < 0.0001; ns—non-significant.

Our results indicate that treatment with THC positively impacted the reduced levels of the Pdx-1 gene and protein, as well as NKX6.1 and FOXO1 proteins, while decreasing the levels of Slc2A2. Treatment with 5 μM CBD restored the reduced levels of the NeuroD1 gene and NKX6.1 and FOXO1 proteins but exhibited a reductive effect on PDX-1 protein levels. THCV further decreased the levels of Pdx-1, MafA, and Slc2A2 mRNA, as well as PDX-1 protein, while showing a positive effect on the reduced levels of the NeuroD1 gene and FOXO1 proteins. CBC increased the reduced levels of NKX6.1 and PDX-1 proteins but further decreased the levels of MafA, Slc2A2, and NeuroD1 mRNA, along with both FOXO1 gene and protein. Interestingly, CBG increased the levels of all studied mRNAs and proteins except for the Slc2A2 gene and FOXO1 protein (Figure 5).

## 3. Discussion

In the current study, the impact of 5 μM of THC, CBD, THCV, CBC, and CBG on the maintenance and function of INS-1 β-cells was evaluated. The selection of the 5 μM concentration is based on previous research [12,13]. For example, our earlier findings demonstrated that 5 μM of THC, CBD, THCV, and CBC exerts mitigatory effects on elevated levels of pro-inflammatory genes and proteins without negatively affecting cell viability [13,14].

### 3.1. The Impact of the Phytocannabinoids on HGHL-Induced Apoptosis in INS-1 β-Cells

HGHL conditions, commonly associated with obesity and insulin resistance, adversely affect the maintenance and function of β-cells by promoting β-cell loss, dysfunction, and dedifferentiation, and thereby the onset of T2DM [15,16]. Accordingly, HGHL conditions induce apoptosis in β-cells by increasing oxidative stress and promoting mitochondrial dysfunction, leading to cellular damage and inflammation. These conditions also activate pro-apoptotic pathways, such as TXNIP upregulation and ER stress, contributing to β-cell dysfunction and loss [17,18]. P-STAT-1 plays a significant role in the regulation of apoptosis in β-cells. In this line, it has been found that P-STAT1, upregulated by HGHL, triggers key pathways in β-cells: dedifferentiation, apoptosis through DP5 induction, and islet inflammation [19].

Our results show that all five phytocannabinoids significantly mitigate HGHL-induced apoptosis in INS-1 β-cells, as evidenced by the apoptosis assay and the response of several apoptotic biomarkers to the phytocannabinoids in HGHL-induced INS-1 β-cells (Figure 1a,b). We also found that treatment with all five phytocannabinoids significantly modulates the elevated TXNIP levels in HGHL-induced INS-1 β-cells (Figure 4), suggesting that TXNIP could be an upstream target for the phytocannabinoids’ mitigatory effects on HGHL-induced apoptosis. In line with our findings, it has been found that in STZ-injected mice, abnormal cannabidiol (Abn-CBD) reduced circulating proinflammatory cytokines and alleviated islet inflammation by decreasing intra-islet TXNIP levels [20]. Furthermore, we demonstrated that THC, THCV, and CBG modulate the elevated levels of P-STAT1 in HGHL-induced INS-1 β-cells (Figure 4). Considering the role of HGHL-induced P-STAT1 in driving inflammation and apoptosis [19], the ability of THC, THCV, and CBG to modulate increased P-STAT1 in HGHL-induced INS-1 β-cells provides another potential mechanism by which these phytocannabinoids exert their modulatory effects on HGHL-induced apoptosis. As previously demonstrated, treatment with THCV and CBN modulates the elevated P-STAT-1 levels in LPS-induced THP-1 cells [14]. Despite the modulatory impact of CBG on apoptosis and the increased levels of C-Caspase-3, C-Caspase-7, Bim_EL_, and Bim_L_, this phytocannabinoid also elevated the levels of BAX and C-PARP (Figure 1). The pattern of these increases is comparable to CBG’s effect on the elevated levels of TXNIP (Figure 4), suggesting that TXNIP may be an upstream target for CBG in the regulation of BAX and C-PARP.

Our results are supported by several studies highlighting the role of phytocannabinoids in controlling ROS, dative stress, and thereby apoptosis. For example, THC has been shown to reduce cardiovascular complications in STZ-induced diabetic rats, likely through its ability to alleviate hyperglycemia, hyperlipidemia, and oxidative stress, rather than directly affecting cannabinoid receptors in cardiovascular cells [21]. Similarly, CBD has been found to have significant potential in improving macrovascular complications associated with diabetes, likely by reducing intracellular oxidative stress and inflammation, thereby preventing cell damage and fibrosis [22,23]. Additionally, CBD has shown promise in improving diabetic retinopathy by addressing both oxidative stress and inflammation [24]. CBG, with its anti-inflammatory effects, has been shown to reduce ROS-induced apoptosis in macrophages [25] and alleviate conditions like inflammatory bowel disease, which suggests its potential for treating diabetes and its associated complications [26].

### 3.2. The Impact of Phytocannabinoids on Cell Cycle Modification in INS-1 β-Cells

HGHL conditions disrupted the cell cycle distribution in INS-1 β-cells, reducing the G1 phase while increasing the S and G2 phases, indicative of accelerated cell cycle progression that may lead to cellular stress and dysfunction [27] (Figure 2). Phytocannabinoids exhibited differential modulatory effects on these alterations: CBC partially restored the G1 phase, suggesting a potential role in promoting cell cycle arrest or stabilization to counteract HGHL-induced proliferation; CBD non-significantly reduced the elevated S phase, indicating a subtle attempt to modulate DNA synthesis processes, while other phytocannabinoids further increased the S phase, potentially reflecting a distinct influence on cell cycle progression under metabolic stress; and THC, THCV, CBC, and CBG reduced the elevated G2 phase, highlighting their potential to regulate cell cycle checkpoints and prevent genomic instability. The G2 phase is a critical period in the cell cycle where cells prepare for mitosis by repairing DNA damage and ensuring genomic integrity. Proper functioning of the G2/M checkpoint is essential to prevent cells with damaged DNA from entering mitosis, thereby maintaining genomic stability. Dysregulation of the G2-phase checkpoint recovery pathway can lead to reduced DNA repair efficiency during the S and G2 phases, resulting in increased genomic instability [28,29,30]. These effects may be attributed to the interaction of phytocannabinoids with ECS, which plays a critical role in regulating β-cell functions, including proliferation and apoptosis [31,32]. By targeting the ECS and its associated signaling pathways, phytocannabinoids may help restore normal cell cycle dynamics disrupted by HGHL conditions, thereby supporting β-cell health and function. Further studies are required to elucidate the precise molecular mechanisms underlying these effects. It is important to note that INS-1 β-cells are cancerous in nature, and their cell cycle dynamics differ from those of normal β-cells. Therefore, to validate our findings, it is essential to replicate the experiments using normal β-cells.

### 3.3. The Impact of Phytocannabinoids on the Impaired Function of HGHL-Induced INS-1 β-Cells

This study demonstrates the diverse effects of phytocannabinoids on GSIS and β-cell functionality in HGHL-induced INS-1 β-cells (Figure 3). Among the phytocannabinoids tested, THC, THCV, CBC, and CBG significantly restored the impaired GSIS. This effect may be attributed to their ability to modulate the elevated levels of TXNIP (Figure 4), thereby reducing oxidative and endoplasmic reticulum (ER) stress—both of which are well-documented contributors to β-cell dysfunction under glucolipotoxic conditions [18,33,34]. The restorative impact of THC, THCV, and CBG on impaired β-cell function may, in part, be attributed to their modulatory effects on P-STAT-1 (Figure 4). As previously mentioned, the upregulation of P-STAT-1 is associated with β-cell loss and dysfunction [19]. Interestingly, despite the modulatory effects of CBD on TXNIP (Figure 4) and apoptosis (Figure 1) in HGHL-induced INS-1 β-cells, CBD negatively impacted GSIS (Figure 3). This may be attributed to its inhibitory effect on the expression of *Ins* genes (Figure 3) and the *Slc2A2* gene, as well as its downregulation of upstream transcription factors such as *MafA* (Figure 5).

Interestingly, although CBD is well-recognized for its anti-inflammatory properties, which could theoretically protect β-cell function, our findings reveal mixed—and in some cases, negative—effects on GSIS. This paradox may be explained by CBD’s complex dual actions on calcium signaling pathways that are central to β-cell function. Beyond its interaction with classical cannabinoid receptors (CB1 and CB2), CBD modulates other molecular targets, including TRPV1 channels, GPR55, and PPARs, each capable of influencing β-cell activity in a context-dependent manner [35]. Notably, CBD’s activation of TRPV1 channels increases intracellular calcium, which initially promotes insulin secretion. However, prolonged or excessive activation can lead to TRPV1 desensitization and calcium dysregulation, impairing GSIS, particularly under HGHL conditions [36]. Interestingly, some evidence suggests that at physiologically relevant low doses, CBD may actually inhibit TRPV1 signaling by suppressing the adenylyl cyclase–cAMP pathway and promoting calcineurin-mediated inhibition—a mechanism known to desensitize nociceptors and potentially reduce calcium influx in β-cells, thereby dampening insulin secretion [37]. Additionally, CBD’s inhibitory effects on other calcium channels may contribute to its mixed impact on GSIS. It has been shown to block T-type calcium channels, important for regulating membrane excitability and supporting insulin release [38]. Moreover, based on findings from cardiomyocyte studies, CBD can inhibit L-type Ca^2+^ channels (Cav1.2 and Cav1.3), which play a crucial role in mediating calcium entry and triggering insulin granule exocytosis in β-cells [39,40]. Collectively, these mechanisms may underlie the impaired insulin secretion observed in our study.

The KSIS test is used to assess the secretory activity of β-cells by stimulating insulin release through elevated potassium levels. The test typically involves increasing extracellular potassium concentrations, leading to membrane depolarization, the opening of voltage-gated calcium channels, and ultimately insulin secretion. KSIS bypasses glucose metabolism and directly triggers insulin release through membrane depolarization, whereas GSIS involves the complete insulin secretion pathway, including glucose metabolism and calcium influx [41,42,43]. According to our results, among the five phytocannabinoids tested, only CBG restored the impaired KSIS in HGHL-induced INS-1 β-cells (Figure 3). This suggests that the positive effect of CBG on β-cell secretory activity may partly contribute to its restoration of impaired GSIS in HGHL-induced INS-1 β-cells. In this study, we also assessed basic insulin production in HGHL-induced INS-1 β-cells. Our results indicated that all tested phytocannabinoids, except CBD, significantly increased basal insulin secretion, which corresponds with their effects on *Ins2* gene expression (Figure 3). This suggests that THC, THCV, CBC, and CBG may enhance basal insulin production and secretion by upregulating the *Ins2* gene in HGHL-induced INS-1 β-cells. It is important to note that none of the phytocannabinoids showed a positive effect on the reduced levels of the *Ins1* gene (Figure 3). The response of the *Ins* genes to the phytocannabinoids was also evaluated in a posttreatment study. Our findings revealed that treatment with THC, CBD, and THCV significantly restored the reduced levels of both *Ins* genes, while CBC and CBG did not upregulate these genes.

### 3.4. The Effects of Phytocannabinoids on the Expression of β-Cell-Enriched Genes and Proteins

In this study, we examined the effects of phytocannabinoids on the expression of key β-cell-specific genes and proteins involved in insulin secretion and β-cell function. Our results demonstrate that THC, CBD, THCV, CBC, and CBG have diverse and distinct impacts on the regulation of these genes and proteins, which likely contribute to their effects on β-cell functionality under HGHL-induced conditions.

The positive impact of THC on *Pdx-1* mRNA, NKX6.1, and FOXO1 proteins (Figure 5) suggests that it may enhance β-cell function through the activation of essential transcription factors that promote insulin gene expression, and β-cell survival and maintenance. CBD, on the other hand, appeared to restore the levels of the *NeuroD1* gene and NKX6.1 and FOXO1 proteins, but it negatively impacted PDX-1 protein levels (Figure 5). NeuroD1 and FOXO1 are critical for β-cell differentiation and survival, and the downregulation of PDX-1 could have a counteracting effect on insulin production (Figure 3). This suggests that CBD might induce a complex regulation of β-cell gene expression. THCV exhibited a distinct pattern of action, as it decreased *Pdx-1*, *MafA*, and *Slc2A2* mRNA levels while enhancing NeuroD1 and FOXO1 protein levels (Figure 5). The positive effect on NeuroD1 and FOXO1 suggests that THCV may also have protective effects on β-cell survival. CBC treatment led to an increase in NKX6.1 and PDX-1 proteins but further decreased the levels of *MafA*, *Slc2A2*, and *NeuroD1* mRNA, along with both FOXO1 gene and protein. These findings suggest that CBC may help restore certain aspects of β-cell function (through NKX6.1 and PDX-1), such as insulin production (Figure 3). Interestingly, CBG showed a broad stimulatory effect on the expression of all studied genes and proteins, except for Slc2A2 and FOXO1. The ability of CBG to enhance the levels of Pdx-1, NKX6.1 (Figure 5), and other transcription factors suggests that it may have a comprehensive effect on improving β-cell function and maintenance under HGHL stress, potentially by upregulating insulin gene expression and promoting β-cell survival pathways.

CB1R plays a crucial role in β-cell function, primarily by regulating insulin secretion and β-cell survival. In β-cells, CB1R activation modulates GSIS, where low CB1R activity is typically associated with improved insulin release. CB1R also influences β-cell apoptosis and inflammation under stress conditions, such as HGHL exposure [11,31,44,45,46]. In the current research, HGHL conditions decreased CB1R expression in INS-1 β-cells, but co-treatment with THC, CBD, and CBC restored CB1R levels (Figure 4), suggesting their potential in protecting β-cell function under metabolic stress. In contrast, THCV and CBG did not affect CB1R expression (Figure 4), indicating that their therapeutic actions may not involve direct modulation of CB1R. These findings highlight the importance of specific phytocannabinoids in regulating the endocannabinoid system to support β-cell health in diabetes.

## 4. Materials and Methods

### 4.1. Chemicals and Reagents

THC (6465-30-1), CBD (13956-29-1), THCV (T-094-1ML), CBC (20675-51-8), CBG (25654-31-3), L-Glutamine (TMS-002-C), sodium pyruvate (S8636), HEPES (TMS-003-C), β-mercaptoethanol (ES-007), and INS-1 832/13 Rat Insulinoma Cell (SCC207) were purchased from MilliporeSigma Canada Ltd., (Oakville, ON, Canada). Roswell Park Memorial Institute Medium (RPMI-1640) (350-060-CL), HBSS1X (311-515), and D-PBS, 1X(311-010-CL) were acquired from Wisent Inc. (Saint-Jean-Baptiste, QC, Canada).

### 4.2. Cell Culture and Treatments

INS-1 832/13 Rat Insulinoma Cells were cultured in RPMI 1640 medium supplemented with 2 mM L-Glutamine, 1 mM sodium pyruvate, 10 mM HEPES, 0.05 mM β-mercaptoethanol, and 10% fetal bovine serum (FBS) ((10082147) acquired from Fisher Scientific Company, Ottawa, ON, Canada), along with 2.5 mM glucose. Cells within passages 5–10 were used for all experiments. After being cultured in a medium containing 2.5 mM glucose for two days, cells were exposed to a specified dosage of individual phytocannabinoids and/or an equal quantity of the vehicle (methanol) for a duration of 2 h. Subsequently, high-glucose (HG) (25 mM glucose) and high-lipid (HL) (400 μM palmitic acid) conditions were introduced for the next 48 h [27,47]. Following the experimental treatments, RNA and protein were extracted from the treated cells to perform quantitative real-time polymerase chain reaction (qRT-PCR) and Western blot analysis, respectively.

Following the experimental treatments, the cells were subjected to a range of assays, including MTT, apoptosis, cell cycle analysis, GSIS, KSIS, Western blotting, and qRT-PCR. For the posttreatment study, the cells were initially cultured in HGHL conditions for 48 h. Following this, the HGHL medium was replaced with standard culture medium containing the specified concentrations of each phytocannabinoid and/or an equivalent amount of methanol, and the cells were incubated for an additional 48 h.

### 4.3. MTT

The effect of five phytocannabinoids on β-cell viability under high-glucose and high-lipid (HGHL) conditions was assessed using the MTT assay. Cells were plated at a density of 5 × 10^4^ cells per well in 100 μL of culture medium containing 5 μM of each phytocannabinoid or an equivalent volume of vehicle (methanol) for 2 h. It is important to note that one of the main objectives of this study was to compare the effects of both minor and major phytocannabinoids under identical experimental conditions, which necessitated the use of a single standardized concentration. The 5 μM dose was chosen based on our optimization experiments and previously published studies, where it produced relevant biological effects without inducing cytotoxicity [11,12,13]. Subsequently, the cells were exposed to medium containing phytocannabinoids, vehicle, and/or HG (25 mM glucose) with HL (400 μM palmitic acid) for 48 h at 37 °C in a 5% CO_2_ environment.

Following incubation, 10 μL of the MTT labeling reagent (3-(4,5-dimethylthiazol-2-yl)-2,5-diphenyltetrazolium bromide; product number 11465007001, MilliporeSigma Canada Ltd., Oakville, ON, Canada) was added to each well, and the plate was incubated for 4 h in a humidified atmosphere (37 °C, 5% CO_2_). To dissolve the resulting formazan crystals, 100 μL of solubilization solution was added to each well, and the plate was left overnight in the humidified incubator. Absorbance was recorded at 595 nm using a microplate reader (FLUOstar Omega, BMG LABTECH, Offenburg, Germany).

### 4.4. Apoptosis Assay

To evaluate apoptosis, the BD Pharmingen™ FITC Annexin V Apoptosis Detection Kit II (Cat No. BDB556570; BD Biosciences, Mississauga, ON, Canada) was employed to detect the externalization of phosphatidylserine (PS) on the cell membrane. FITC Annexin V specifically binds to PS exposed on apoptotic cells, while propidium iodide (PI) helps differentiate live cells from those that are necrotic or late-apoptotic. Cells labeled only with FITC Annexin V were categorized as apoptotic, those positive for PI were identified as necrotic or in late stages of apoptosis, and cells negative for both dyes were considered viable. To begin, cells were rinsed twice with cold D-PBS and resuspended in 1× Annexin V Binding Buffer at a concentration of 1 × 10^6^ cells/mL. A 100 µL sample (containing 1 × 10^5^ cells) was transferred to a 5 mL tube, and 5 µL of FITC Annexin V, and with 5 µL of PI were added. The mixture was gently mixed and incubated in darkness at room temperature (approximately 25 °C) for 15 min. Following incubation, 400 µL of 1× Binding Buffer was added to the tube, and the samples were analyzed within one hour, using a BD FACSAria™ Fusion Flow Cytometer (BD Biosciences, San Jose, CA, USA).

### 4.5. Cell Cycle Assay

Cells were collected, rinsed with PBS, and fixed by gently adding cold 70% ethanol to the cell pellet while continuously mixing to avoid clumping. The cells were then incubated at 4 °C for 30 min to complete the fixation process. Following fixation, the cells were washed twice with PBS via centrifugation at 850 g, carefully removing the supernatant to minimize sample loss. To eliminate RNA, 50 µL of RNase solution (100 µg/mL) was added to each sample, followed by the addition of 200 µL of a propidium iodide (PI) solution prepared at 50 µg/mL to stain the DNA. Samples were then incubated for 30 min at room temperature in the dark. DNA-bound PI fluorescence was measured using the BD FACSAria™ Fusion Flow Cytometer (BD Biosciences), providing high-resolution fluorescence detection for accurate analysis of PI signals during flow cytometry.

### 4.6. Western Blotting

The cells were washed twice with cold PBS to remove debris and then lysed with RIPA buffer to extract proteins. The lysates were centrifuged at 13,000 rpm for 15 min, and the supernatant containing proteins was collected in new microtubes. Protein concentration was determined using the Bradford assay, ensuring accurate quantification before Western blot analysis. Proteins were separated using polyacrylamide gels with varying concentrations (8–12%) and subsequently transferred onto PVDF membranes (Amersham Hybond^®^ P, RPN2020F, GE Healthcare, Chicago, IL, USA). Blocking was performed using PBST containing 1% Tween-20 and 5% milk to prevent non-specific binding, followed by overnight incubation at 4 °C with primary antibodies.

After three PBST washes, the membranes were incubated with secondary antibodies for two hours at room temperature, followed by another series of PBST washes. Immunoreactive bands were visualized using peroxidase-conjugated secondary antibodies and the ECL Plus Western Blotting Detection System (GE Healthcare). Band intensities were quantified using ImageJ 1.54 K software and normalized to the corresponding housekeeping protein. The primary antibodies used in this study included markers such as Cleaved Caspase-3 (Asp175, Product No. 9664), Cleaved Caspase-7 (Asp198, Product No. 8438), Cleaved PARP (Asp214, Product No. 5625), Bim (Product No. 2933), Bax (Product No. 2772), PDX-1 (Product No. 5679), FoxO1 (Product No. 2880S), NKX6.1 (Product No. 54551), and TXNIP (Product No. 14716S), all sourced from Cell Signaling Technology (Whitby, ON, Canada). Additional antibodies, such as Anti-P-STAT1 (phospho S727, Cat No. ab109461), Anti-CB1R (Cat No. ab259323), Anti-α-Tubulin (ab7291), and Anti-β Actin (Cat No. ab8227), were acquired from Abcam (Toronto, ON, Canada). Secondary antibodies were obtained from cell signaling technology (Whitby, ON, Canada).

### 4.7. qRT-PCR

Total RNA was extracted using TRIzol™ reagent (Invitrogen, Life Technologies Inc., Burlington, ON, Canada), and its concentration was measured using a Nanodrop spectrophotometer (NanoDrop 2000c, ThermoFisher Scientific, Waltham, MA, USA). For cDNA synthesis, 1 μg of RNA was reverse-transcribed using the iScript™ Reverse Transcription Supermix (BioRad Laboratories, Saint-Laurent, QC, Canada). The synthesized cDNA was used as a template in quantitative PCR (qPCR), with 1 μL of cDNA per reaction. The qPCR was conducted with SsAdvanced™ Universal Inhibitor-Tolerant SYBR Green Supermix (Bio-Rad Laboratories, Saint-Laurent, QC, Canada). The primers were designed based on previously published sequences [27,47].

### 4.8. Glucose-Stimulated Insulin Secretion (GSIS) and Potassium-Stimulated Insulin Secretion (KSIS)

To evaluate β-cell function under HGHL conditions, GSIS and KSIS assays were performed. Cells were seeded in 24-well plates at 0.5 × 10⁴ per well, incubated for 2 days, and pretreated with the appropriate treatment concentrations for 2 h before exposure to HGHL conditions for 48 h. Following treatment, cells were washed with HEPES Balanced Salt Solution (HBSS) containing 2.5 mM glucose and incubated for an additional hour in the same solution. Three experimental conditions were tested: normal glucose (2.5 mM), high glucose (16.5 mM), and glucose plus KCl (50 mM). After 2 h of incubation, insulin secretion was measured using the Rat/Mouse Insulin ELISA Kit (EZRMI-13K, Sigma Aldrich). For the assay, 10 μL of the sample and 80 μL of Detection Antibody were added to each well, followed by a 2 h incubation. After washing, Enzyme Solution was added, and the reaction was stopped after 30 min. Substrate Solution was then added, and absorbance was measured at 450 nm and 590 nm, using a SpectraMax i3x Multi-Mode Microplate Reader (Molecular Devices, San Jose, CA, USA). Samples were diluted 150-fold for GSIS and KSIS assays to ensure accurate readings within the assay’s detection range.

### 4.9. Statistics

The data were subjected to statistical analysis, using one-way ANOVA to assess differences between groups. Post hoc comparisons were performed using Duncan’s multiple range test and Tukey’s test to further evaluate group mean differences. All analyses were conducted using GraphPad Prism version 9.5.1 (GraphPad Software, San Diego, CA, USA). Results are expressed as mean ± standard deviation (SD), with statistical significance determined at a *p*-value less than 0.05.

## 5. Conclusions

Our findings demonstrate that all five phytocannabinoids tested effectively mitigate high-glucose–high-lipid (HGHL)-induced apoptosis in INS-1 β-cells, primarily through their mitigatory effects on thioredoxin-interacting protein (TXNIP). Among the tested compounds, THC exhibited the most pronounced impact on reducing TXNIP levels and apoptotic biomarkers, suggesting that THC may be the most promising candidate for counteracting oxidative stress and apoptosis in HGHL-induced β-cells.

Additionally, all phytocannabinoids except CBD were able to restore impaired glucose-stimulated insulin secretion (GSIS) in HGHL-induced β-cells. Based on the observed trends, THC and CBC demonstrated the most significant potential to improve GSIS, positioning them as strong candidates for further investigation in the context of β-cell function under diabetic-like conditions.

The effects of the phytocannabinoids on the expression of β-cell-specific genes and proteins revealed varied responses. Notably, CBG was particularly effective in restoring the expression of multiple genes and proteins critical for the maintenance and functionality of β-cells. This highlights CBG’s potential as a valuable compound in preserving β-cell identity and function in the face of metabolic stress.

While these findings underline the therapeutic promise of phytocannabinoids in protecting β-cells from metabolic stress, further studies are required to validate these effects in in vivo models. Additionally, exploring the long-term safety, efficacy, and mechanisms of action of these compounds will be critical for advancing their potential clinical applications in diabetes management.

## 6. Limitations and Future Directions

The most significant limitation of the current study is the absence of in vivo studies. While the findings provide valuable insights into the effects of phytocannabinoids on β-cells under high-glucose and high-lipid conditions, they remain limited to in vitro models. Future research should extend these investigations to primary human islets or human stem cell-derived β-cells and in vivo studies to better understand the long-term physiological relevance and therapeutic potential of these compounds in animal models and, eventually, clinical settings.

Additionally, this study focused exclusively on CB1R as the primary receptor of the endocannabinoid system. To achieve a more comprehensive understanding, future studies should explore the responses of other endocannabinoid system components to phytocannabinoids, including CB2R, GPR55, and the enzymes involved in endocannabinoid metabolism (e.g., FAAH and MAGL). Investigating these components may reveal novel mechanisms and broaden our understanding of the endocannabinoid system’s role in β-cell function and survival.

Another limitation lies in the restricted scope of β-cell dedifferentiation markers studied in this research. While we examined specific β-cell transcription factors and genes/proteins associated with dedifferentiation, we did not address other critical aspects, such as epigenetic modifications, chromatin remodeling, and changes in DNA methylation or histone acetylation patterns. Future research should incorporate these epigenetic dimensions to provide a more holistic understanding of the dedifferentiation process and its reversibility under different treatment regimens.

## Data Availability

All raw Western blot images were uploaded to the journal.
